# Retraction Note: Polydatin improves osteogenic differentiation of human bone mesenchymal stem cells by stimulating TAZ expression via BMP2-Wnt/β-catenin signaling pathway

**DOI:** 10.1186/s13287-023-03412-6

**Published:** 2023-07-25

**Authors:** Ying-Shan Shen, Xiao-Jun Chen, Sha-Na Wuri, Fan Yang, Feng-Xiang Pang, Liang-Liang Xu, Wei He, Qiu-Shi Wei

**Affiliations:** 1 First Clinical Medical College, Guangzhou University of Chinese Medicine, Guangzhou, Guangdong China; 2 Key Laboratory of Orthopaedics & Traumatology, The First Affiliated Hospital of Guangzhou University of Chinese Medicine, Guangzhou University of Chinese Medicine, Guangzhou, China; 3 Hip Preserving Ward, No. 3 Orthopaedic Region, The First Affiliated Hospital of Guangzhou University of Chinese Medicine, Guangzhou, Guangdong China; 4 No. 3 Orthopaedic Region and Institute of the Hip Joint, The First Affiliated Hospital of Guangzhou University of Chinese Medicine, Guangzhou, Guangdong China; 5 Third Clinical Medical College, Guangzhou University of Chinese Medicine, Guangzhou, Guangdong China; 6 The Third Affiliated Hospital of Guangzhou University of Chinese Medicine, Guangzhou, Guangdong China; 7 Institute of orthopedics of Guangzhou University of Chinese Medicine, Guangzhou, Guangdong China

**Retraction Note : Stem Cell Research & Therapy (2020) 11:204** 10.1186/s13287-020-01705-8

The authors have retracted this article. While trying to replicate this study they found that the polydatin could not recover the mRNA and protein expression of RUNX2, Osteopontin, DLX5, and OCN after lentivirus transfection. The authors believe that this could be due to contamination of lentivirus or the vector, or that polydatin was wrongly prepared in this study. All authors agree to this retraction.

